# Allografts of the Acellular Sciatic Nerve and Brain-Derived Neurotrophic Factor Repair Spinal Cord Injury in Adult Rats

**DOI:** 10.1371/journal.pone.0042813

**Published:** 2012-08-28

**Authors:** Changyu Li, Xiangtong Zhang, Ronglong Cao, Bohai Yu, Hongsheng Liang, Min Zhou, Dayong Li, Yuehua Wang, Enzhong Liu

**Affiliations:** 1 Key Laboratory in Cell Transplantation in Ministry of Health of China, Department of Neurosurgery, The First Affiliated Hospital of Harbin Medical University, Harbin, China; 2 Immunity and Infection, Pathogenic Biology Key Laboratory, Department of Microbiology, Harbin Medical University, Heilongjiang Province, Harbin, China; 3 Department of Pathology, The First Affiliated Hospital of Harbin Medical University, Harbin, China; 4 The Second Affiliated Hospital of Harbin Medical University, Harbin, China; University of Pittsburgh, United States of America

## Abstract

**Objective:**

We aimed to investigate whether an innovative growth factor-laden scaffold composed of acellular sciatic nerve (ASN) and brain-derived neurotrophic factor (BDNF) could promote axonal regeneration and functional recovery after spinal cord injury (SCI).

**Methods:**

Following complete transection at the thoracic level (T9), we immediately transplanted the grafts between the stumps of the severed spinal cords. We evaluated the functional recovery of the hindlimbs of the operated rats using the BBB locomotor rating scale system every week. Eight weeks after surgery, axonal regeneration was examined using the fluorogold (FG) retrograde tracing method. Electrophysiological analysis was carried out to evaluate the improvement in the neuronal circuits. Immunohistochemistry was employed to identify local injuries and recovery.

**Results:**

The results of the Basso-Beattie-Bresnahan (BBB) scale indicated that there was no significant difference between the individual groups. The FG retrograde tracing and electrophysiological analyses indicated that the transplantation of ASN-BDNF provided a permissive environment to support neuron regeneration.

**Conclusion:**

The ASN-BDNF transplantation provided a promising therapeutic approach to promote axonal regeneration and recovery after SCI, and can be used as part of a combinatory treatment strategy for SCI management.

## Introduction

Spinal cord injury (SCI) is a disaster for humans. There are many obstacles that hamper the healing of SCI. One of the most important obstacles lies in the ‘hostile’ microenvironment, which could inhibit axonal regeneration [1]. But several research have recently demonstrated that mammalian CNS axons could regenerate in a proper environment [2]–[4]. Such approaches were mainly focused on providing supportive substrates and neurotrophic factors to injured axons. Nevertheless, an ideal scaffold material has yet to be developed.

Peripheral nerve grafts (PNG) provide a growth-permissive substratum for local neurotrophic factors to enhance the regeneration of axotomized neurons when grafted into the site of spinal cord injury [5]–[8].

Acellular nerve allograft has been used to repair the peripheral nerve gap with excellent efficacy, and thus is a compelling candidate material to bridge the peripheral nerve gap and provide a favorable local environment to regenerating axons [9]. Therefore, it would be interesting to make certain whether acellular nerve allografts could promote axonal regeneration and functional recovery when transplanted into the lesion site of the spinal cord. The sciatic nerve as the thickest peripheral nerve in the body has a rich extracellular matrix. So we chose to make the scaffold based on sciatic nerve.

BDNF plays crucial roles in the physiology of developing and mature nervous systems, and is necessary for the survival and function of neurons. BDNF has also been shown to play an important role in modulating functional neuroplasticity following SCI via the activation of the TrkB [10].

One innovative way to repair SCI is to bridge the lesion sites with a growth factor-laden scaffold. Based on the ability of BDNF to support nerve growth and the characteristics of the ASN, we hypothesized that the growth factor-laden scaffold could be directly transplanted into the damaged spinal cord. The aim of this work was to assess the efficacy of the combination of ASN and BDNF (ASN-BDNF) in the repair of SCI.

## Results

### Basal Lamina Staining of the ASN

**Figure 1:**
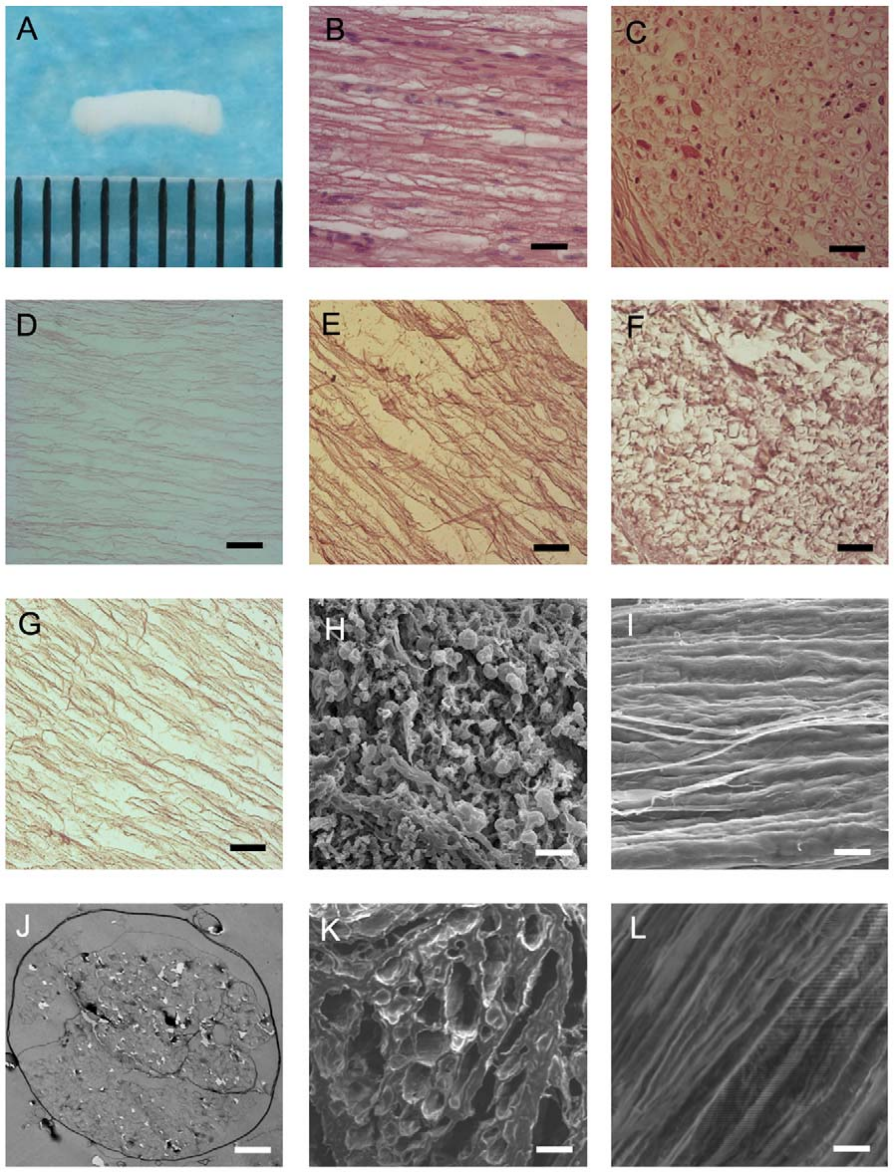
Basal Lamina Staining of the ASN and Morphology of ASN. (A): the sciatic nerve after extraction. (B,D): hematoxylin-eosine (H&E) staining of fresh nerve and nerve segment after the extraction procedure in longitudinal section. (C) H&E staining of fresh nerve in transection. (E): immunohistochemical staining of collagen type III. (F): immunohistochemical staining of collagen type I. (G): immunohistochemical staining of laminin. Scale bars = 200 µm(B,C,D,E,F,G). SEM image of fresh nerve in transection (H) and in longitudinal section(I). SEM image of the same nerve after the extraction procedure in transection (K) and in longitudinal section(L). Scale bars = 20 µm(H,K),10 µm(I,L). (J)TEM image of ASN: the cells and myelin Sheath diepeared entirely. Scale bars = 2 µm(J).

After extraction, the nerve turned soft and rarefaction(Fig. 1A). Histological analysis was used to assess the extraction of cellular components and the intact extracellular matrix was obtained. H&E staining of a fresh nerve segment in longitudinal section (Fig. 1B) showed many cells and nucleus spreading along the axons, and the very same staining of ASN (Fig. 1D) clearly revealed that the extraction procedure had completely removed the cells from individual nerve fibers, leaving only empty basal laminatubes. HE staining of fresh nerve in transection displayed many axons and schwann cells (Fig. 1C), while extracted nerve section (Fig. 1F) indicated polyporous structures without axons and cells. Staining for the presence of laminin and collagen type III, type I (Fig. 1E, 1F, 1G) showed that the extracellular matrix components were present in ASN.

### Morphology of the Scaffold

SEM of a piece of fresh nerve (Fig. 1H and 1I) showed a compact construction, but the ASN (Fig. 1K and 1L) presented numerous pores in coronal plane on and parallel grooves in the longitudinal section. Meanwhile, the arrangement of the grooves was orderly, indicating that the structure of ASN was not severely destroyed during the extraction procedure. The SEM image revealed the scaffold had a three-dimensional tubiform structure. According to the SEM, individual channels within the scaffolds had a cross-section diameter of 20–100 µm. TEM of ASN (Fig. 1J) showed the cells and myelin sheath were absent or demyelinated and that apparently empty basal lamina tubes remained in the endoneurium.

### Locomotion Recovery

Hindlimb locomotor recovery was measured using BBB scores in different groups by repeated-measures ANOVA (P>0.05). Spontaneous recovery of hindlimb function occurred in all groups since 3 weeks PO. But there is no significant difference between individual groups (date not shown).

### Electrophysiological Testing

**Figure 2:**
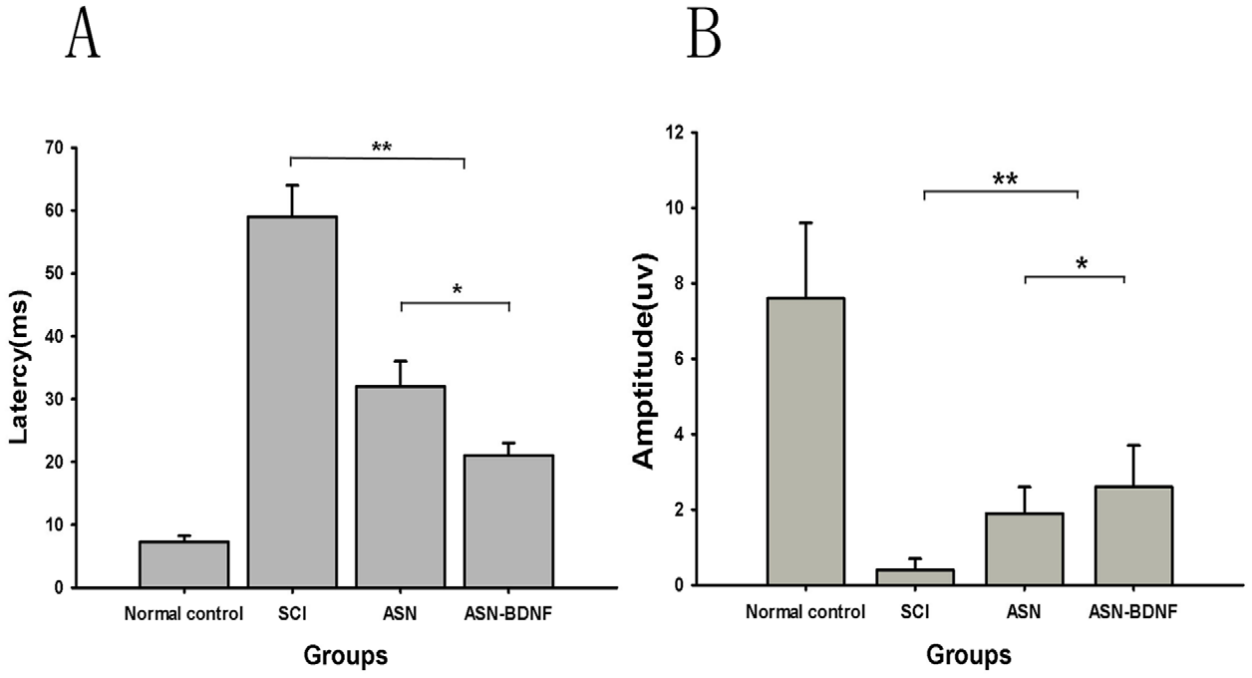
The latency and amplitude for ASN-BDNF group were recovered in the 8th week PO. (A): latency of SEP in different groups in the 8^th^ week PO. (B): amplitude of SEP in different groups in the 8^th^ week PO. The latency and amplitude in ASN-BDNF group were recovered compared with SCI and ASN groups. *P<0.05: ASN-BDNF group vs ASN group, **P<0.01: ASN-BDNF group or ASN group vs SCI group.(ANOVA, SNK q test).

Spinal cord conductivities were measured immediately after the SCI and then 8 weeks PO for each group. No SEP was recorded in rats of all SCI groups at day 1 PO (data not shown). Eight weeks later, both the latency and amplitude of SEP were restored in the ASN-BDNF group (latency: 22.3±1.1 ms; amplitude: 2.83±0.2 µV) compared to the SCI group (latency: 59.0±3.2 ms; amplitude: 0.38±0.15 µV; P<0.01). Similarly, the ASN group also demonstrated some recovery in their electrophysiological examinations (Fig. 2A and 2B). These data indicated that the neuronal circuits were partially reestablished in the transplantation groups, with the best recovery in the ASN-BDNF group.

### Neuroanatomical Retrograde Tracing

**Figure 3:**
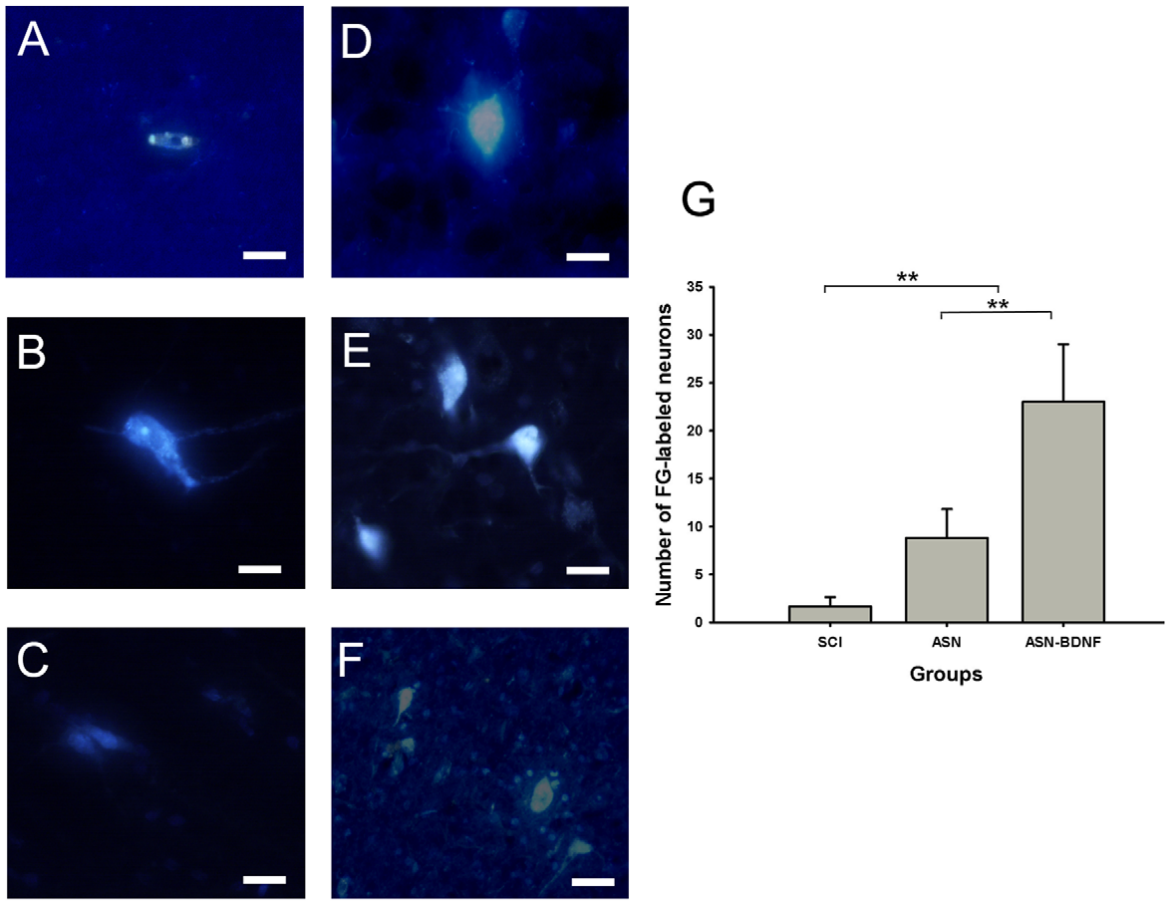
More FG-labeled neurons were detected beyond the transected area in ASN-BDNF group. Fluorogold-labeled neurons within the sensorimotor cortex(A,D), brainstem(B,E) and rostral spinal cord nearby the transected site(C,F) in ASN group(A,B,C) and ASN-BDNF group(D,E,F). Scale bars = 100 µm. (G) Comparison of the number of FG-labeled neurons in T6 spinal segments in different groups. The number in ASN-BDNF group was the highest. **P<0.01: ASN-BDNF group vs ASN group, ASN-BDNF group vs SCI group, ASN group vs SCI group.

In the SCI group, few FG-labeled neurons could be found in the sensorimotor cortex, brainstem, or spinal cord rostral to the transected site. In the ASN and ASN-BDNF groups, more FG-labeled neurons were detected in some sections (Fig. 3A–3F). Especially in the ASN-BDNF group, there were many super brilliant FG-labeled neurons in brain stem sections. The difference in the number of FG-labeled axons in T6 spinal segment between the ASN-BDNF and SCI groups or the ASN group and SCI groups was statistically significant (p<0.01). The difference in the number of FG-labeled axons between the ASN-BDNF and ASN groups is significant (p<0.01) (Fig. 3G). These results were consistent with the SEP consequence.

### Histology and Immunohistochemistry

**Figure 4:**
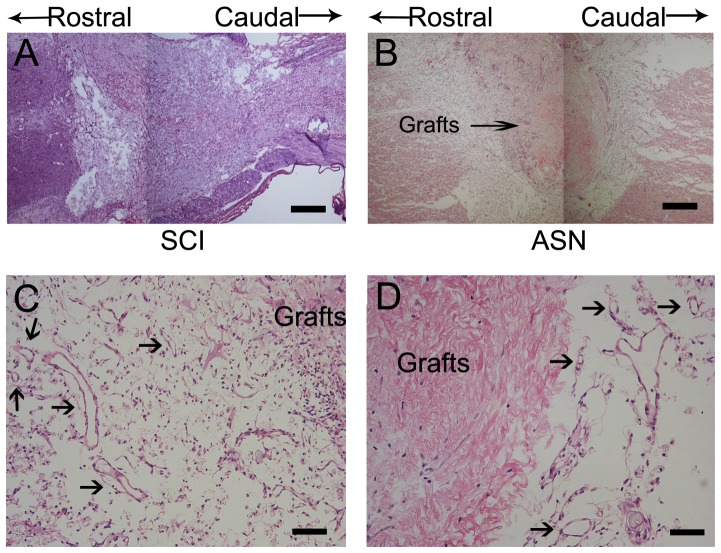
HE staining in longitudinal section of transected area after SCI 8th week PO. HE staining in longitudinal section of transected area from SCI group(A) and ASN group(B). At higher magnification, numerous newly formed blood vessels were recognizable around the ASN grafts(C, D). Scale bars = 200 µm(A,B), 100 µm(C),50 µm(D).

The scars in the SCI animals were characterized by larger and more cavities (Fig. 4A). In the ASN group, cavities were fewer and the ASN grafts had well integrated with the spinal cord (Fig. 4B).We also observed a higher density of microvessels located around the ASN grafts in the ASN group than in the SCI group. Numerous newly formed blood vessels were identified around the ASN grafts (Fig. 4C, 4D).

**Figure 5:**
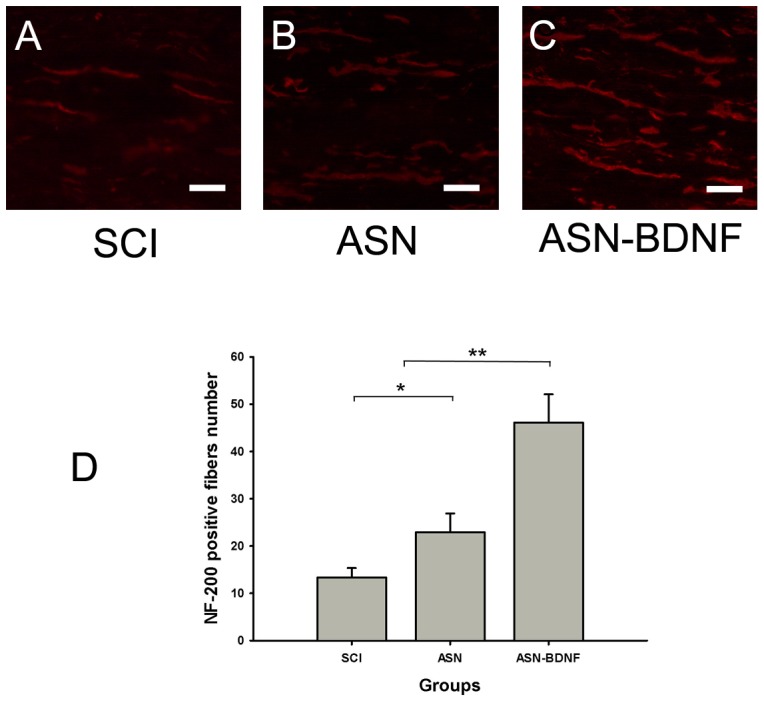
Immunohistochemical staining of NF-200. (A,B,C): Immunohistochemical staining of NF-200 rostral to spinal cord lesion site from SCI group, ASN group and ASN-BDNF group, respectively. Scale bars = 50 µm(A,B,C). (D): Quantification of NF-200 positive axons rostral to the lesion site in different groups. The number in ASN-BDNF group was higher than other groups. *P<0.05: ASN group vs SCI group, **P<0.01: ASN-BDNF group vs ASN group or SCI group. (ANOVA, SNK q test).

While the SCI animals showed few positive fibers (Fig. 5A), an extensive growth of regenerating fascicular nerve fibers was detected in the ASN-BDNF group by the anti-NF-200 immunolabeling from the rostral to spinal cord lesion site (Fig. 5C). We quantified the number of fibers positive for NF-200 within a 500 µm range from the rostal to the lesion site (Fig. 5D), and found that the number of the ASN-BDNF group was significantly higher than for other groups (P<0.01). The difference in the number of axons between ASN and SCI was statistically significant (P<0.05).

## Discussion

Several promising therapies for SCI, which have emerged from the laboratory since the late 1960s, have entered into the stage of prospective randomized clinical trials. However, it is disappointing that few of these therapies have had convincing efficacy in the treatment of SCI [5]. The failure of spontaneous axonal regeneration, which would result in persistent functional impairment within the CNS, can be caused by a number of factors, including a poor intrinsic regenerative capacity of CNS neurons, a ‘hostile’ microenvironment, and a shortage of substance promoting neuron regeneration. In an elegant series of studies, Many studies [3], [11] have showed that adult CNS axons have the potential to regrow after being severed if provided with an appropriate environment. Transplants have been designed to provide a growth-permissive environment to enhance the regenerative effort of CNS neurons. Even a small amount of rescued connections can provide significant functional gain [12]. The axonal growth into a PNG and the formation of synaptic contact with host neurons have been demonstrated by previous studies using different animal models, but growth beyond the PNG is only limited to a small number of axons over a short distance [4], [13], [14]. One important reason lies in the immunological rejection after transplantation. A growing body of evidence have suggested that Schwann cells may modulate local immune responses by recognizing and presenting antigens [15], and cell debris might block axon extension through the channel. Based on a procedure developed by Sondell, we have developed a new method which is composed of freezing, chemical extraction, and mechanical vibration to obtain ASN that is cyto- and immuno-compatitive [3], [16]. Till today, the application of acellular nerve segment in the treatment of SCI has not been studied yet. We believe that the three-dimensional scaffold with relatively ideal mechanical properties, cell-adhesivity, and bio-degradability will provide a promising therapeutic approach to promote axonal regeneration..

We found that the latency and amplitude of SEP were recovered in the ASN-BDNF group compared to the other groups, indicating that axonal circuits were reestablished between the ends of the lesions. Fluorogold can be transported along the axon in a retrograde way, and it has been used as a marker for axonal regeneration. Furthermore, the number of FG-positive neurons should be proportional to the extent of axon regeneration. Obviously, the largest number of FG-positive neurons was found in the ASN-BDNF group, followed by the ASN group, and barely in the SCI group.

We also observed a higher density of microvessels located around the ASN grafts in the ASN group than in the SCI group. This observation indicated that ASN probably can promote angiogenic response and improve local blood supplying. Our results are consistent with a previous study by Ribatti et al. [17], in which acellular brain scaffolds were able to induce a strong angiogenic response when grafted onto the chorioallantoic membrane of chick embryos. Acellular brain scaffolds might induce the release of endogenous angiogenic factors, such as fibroblast growth factor-2 (FGF-2) and vascular endothelial growth factor (VEGF), from the extracellular matrix of the host.

In our opinion, there are some reasons for ASN-BDNF to support axon regeneration and functional recovery. First, the main components of the ASN are collagen and laminins. Collagen type I and III are believed to provide mechanical support for axonal growth and regeneration [18]. Laminin provides attachment sites for cells via cell surface proteins and initiate signals that influence cell behavior and survival, and thus is a good material to benefit nerve restoration. Second, ASN-BDNF could be utilized as part of a trophic factor delivery system with embedded sustained release vehicles. The key point of the repairing is to construct a practical means of not only stimulating, but also guiding the growth of axons across a site of injury. Third, the ASN is degradable [16], which represents less risk of inflammation and nerve compression over time and avoids a second surgery to remove the transplants. At last, the histological results showed that the ASN alone could also be a good material to repair SCI and provided a favorable local environment to regenerate axons, although the axonal regeneration was insufficient and the functional recovery was limited.

In the future research, we will focus on the angiogenic response induced by the ASN and continue to take more precise measures to improve the microenvironment within the lesion sites.

In summary, the data presented in this report demonstrated that ASN-BDNF can benefit the therapeutic treatment of SCI in adult mammals by providing a favorable microenvironment.

## Materials and Methods

### Animals

Adult female Wistar rats weighing 200 g±20 g were purchased from the Laboratory Animal Department of Harbin Medical University and maintained under specific pathogen-free conditions at 21±2°C and 45±5% humidity. All animal procedures were in accordance to guidelines of Ethics Committee of the First Clinical College of Harbin Medical University (201012). All rats used in these studies were bred and maintained in accordance with the guidelines for the Care and Use of Laboratory Animals published by the China National Institute of Health.

### Preparation of the acellular nerve

Wistar rats were killed by overdose anesthesia with Hydral. The sciatic nerves were excised and then dealed with chemical detergents to turn into acellular allografts. The method for the isolation of the ASN was based on a procedure developed by Sondell et al [19]. Briefly, isolated sciatic nerves were immersed in distilled water, and frozen at −80°C for 1 h. Then they were placed into distilled water at room temperature for 6 h. After that these nerves were then exposed to 3% Triton X-100 in distilled water at room temperature with mechanical vibration at 120 r/min for 12 h, followed by a 24 h vibrating period at room temperature in a solution of 4% sodium deoxycholate in distilled water. The extraction procedure was then repeated again. After the nerves were washed with water once every day for the next three days, they were frozen at −30°C and freeze-dried by vacuum sublimation for 5 h, and finally at room temperature until it reached a constant weight. These freeze-dried acellular sciatic nerves were kept at −80°C until use.

### The evaluation of the structure and composition

#### Basal lamina staining

Following being fixed in formalin and embedded in paraffin, sections of 3 µm thickness were obtained and H&E staining was performed.

Furthermore, immunohistochemical staining was performed to examine the presence of collagen and laminin, the basal lamina components of the extracellular matrix (ECM). The primary antibodies used were as follows: (1) polyclonal rabbit anti-rat collagen type I(Santa Cruz Biotechnology, Amerima), 1∶200; (2) polyclonal rabbit anti-rat collagen type III (Santa Cruz Biotechnology, Amerima), 1∶200; (3) polyclonal rabbit anti-rat Laminin(Santa Cruz Biotechnology, Amerima), 1∶200, at 4°C overnight. Secondary antibody staining was performed using an SABC kit. Negative controls were stained similarly, with the primary antibodies replaced with PBS. All slides were counterstained with haematoxylin and mounted before viewing.

#### SEM and TEM analysis

This acellular nerve was frozen at −30°C and freeze-dried by vacuum sublimation for 5 h, and finally at room temperature until it reached a constant weight. The preparations were mounted and sputtered with gold and were observed and photographed using a HITACHI S-3400N SEM.

For transmission electron microscopy (TEM), ASN was fixed in 2.5% glutaraldehyde in 0.1 M PBS, postfixed in 2% osmium tetroxide, dehydrated through a graded ethanol series, and embedded in epoxy resin. Ultrathin (70 nm) sections were collected on copper grids and stained for 1 hour each with uranyl acetate and 1% phosphotungstic acid and for 20 min with Reynolds lead citrate before examination on a transmission electron microscope (JEOL 1200, Japan) [20].

#### Combination of BDNF and ASN

Prior to implantation, 2 mm long segments were cut from ASN following the longitudinal direction of the pores. ASN were rehydrated with 2 µl PBS containing 1 µg BDNF (sigma) protein(a total of 1 µg BDNF per scaffold). For the control scaffolds, the walls were rehydrated with 2 µl PBS [3].

### Evaluation of nerve regeneration

#### Surgery and Experimental Groups

A total of 35 adult female Wistar rats (200±20 g) were used in this experiment. The rats were anaesthetized using an i.p. injection with 10% Hydral (0.3 ml/100 g). After laminectomy was performed at level T9, the dura was opened and the spinal cord was completely transected using iridectomy scissors. A 2 mm craniocaudal section of spinal cord was resected under a surgical microscope. The length of the ASN segment was trimmed to fit against the cross-section of the leision. Two grafts were placed lengthwise into the gap side by side. The wound was closed in layers.

The rats were randomly divided into four groups: a normal control group (laminectomy only, n = 5), a SCI group (no transplantation after SCI, n = 10), an ASN group (ASN transplantation after SCI, n = 10), and an ASN-BDNF group (ASN and BDNF transplantation after SCI, n = 10). After the surgery, the rats were given extensive care.

#### BBB Locomotor Rating Scale

One day PO and weekly thereafter, the rats were functionally monitored using the well-characterized BBB scale [21]. Behavioral tests were performed at the same time every week and graded by the same two observers blinded to treatment conditions.

#### SEP Analysis

Before the rats were sacrificed, they were anesthetized and stereotaxically fixed for cortical SEP analysis. Active recording electrodes were placed over both hindlimb sensory cortices located 2 mm to either side of the midline and 2 mm anterior to the bregma. The reference electrode was anchored epidurally over the olfactory bulb. Stimulating electrodes were placed on each hindlimb and the SEP was recorded on both cerebral hemispheres following right hindlimb stimulations. The bandpass filter setting was 1.9 Hz and each sweep was 0.2 ms. The SEP signals were averaged after 300 stimulation. Waveforms were recorded on a Cadwell Excel machine (S-100, Medtronic, Dantec Company, Denmark).

#### Neuroanatomical Retrograde Tracing

One week before the animals were sacrificed, the spinal cord distal to the injury/implantation site was re-exposed and 5 µl FG (4%)(Fluorochrome, Inc) was injected bilaterally into the spinal cord caudal 10 mm to the transected site and 0.5 mm lateral from the spinal mid-line using a microsyringe.

Animals were killed at 8^th^ week, then we made frozen section in level of T4–T8 in longitudinal plane, brain stem in longitudinal plane and sensorimotor cortex in the coronal. Seven frozen sections were selected at every 100 µm interval. The total number of FG positive neurons in the central 500 µm portion of T6 were counted under the UV filter [22].

#### Histology and Immunohistochemistry

Eight weeks PO following overdose anesthesia, the rats were exsanguinated using the left cardiac ventricle perfusion with 0.9% normal sodium followed by 4% paraformaldehyde in distilled water. The entire spinal cord was obtained, postfixed for 4 h, and dehydrated in 30% sucrose. Sections (6 µm thick) were cut longitudinally with a cryostat for HE staining and immunohistochemistry. The sources and dilution of primary antibodies were mouse anti neurofilament protein-200(1∶200; Santa Cruz Biotechnology, Amerima). For fluorescence, secondary antibodies were Rhodamine conjugated antimouse antibodies (1∶100; Boster, Wuhan, China).

For measurements, seven immunohistochemistry sections were selected at every 100- µm interval. Sprouts just 500 µm rostal to lesion site were counted by viewing enlarged photographs under a fluorescence microscope. The number of sprouts in 21 sections for each group were expressed as means ± standard deviation [22], [23].

#### Statistical Analysis

The BBB scale score was analyzed using repeated-measures ANOVA. Other data were compared between groups using one-way analysis of variance (ANOVA) followed by Student-Newman-Keuls post hoc q test (SNK q test). All the data from this study were presented as a mean with standard deviation (SD), with a value of P<0.05 considered to be significant.
